# 2-{[(*E*)-2-Hy­droxy­benzyl­idene]amino}-1*H*-isoindole-1,3(2*H*)-dione

**DOI:** 10.1107/S1600536810038407

**Published:** 2010-10-02

**Authors:** H. C. Devarajegowda, H. D. Revanasiddappa, L. Shiva Kumar, V. Sumangala, V. D. Jagadeesh Prasad

**Affiliations:** a Department of Physics, Yuvaraja’s College (Constituent College), University of Mysore, Mysore 570 005, Karnataka, India; b Department of Studies in Chemistry, Manasagangotri, University of Mysore, Mysore 570 006, Karnataka, India; c Sequent Scientific India Limited, Baikampadi, Mangalore, Karnataka, India; d Department of Chemistry, Mangalore University, Mangalagangotri 574 199, Karnataka, India

## Abstract

In the title compound, C_15_H_10_N_2_O_3_, the isoindoline ring system is almost planar [maximum deviation = 0.020 (2) Å] and makes a dihedral angle of 1.57 (7)° with the benzene ring. Intra­molecular O—H⋯N and C—H⋯O hydrogen bonds are observed.

## Related literature

Based on the multiple binding sites of acetyl­cholinesterase (AChE), a series of AChE inhibitors involving phthalimide derivatives have been designed and synthesized, see: Zhao *et al.* (2009 ▶). Phthalimide derivatives have also been developed as LXRa-selective antagonists, see: Motoshima *et al.* (2009 ▶). For the biological activity of Schiff bases, see: Singh *et al.* (2006 ▶); Sithambaram *et al.* (2006 ▶); Walsh *et al.* (1996 ▶). For a related structure, see: Jing *et al.* (2007 ▶).
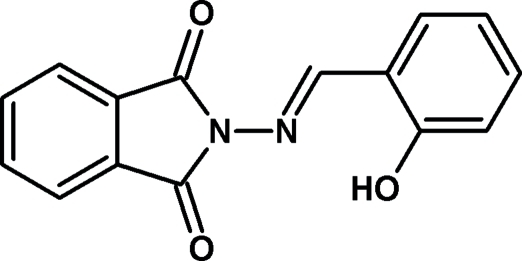


## Experimental

### 

#### Crystal data

C_15_H_10_N_2_O_3_
*M* *_r_* = 266.25
Monoclinic, 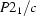

*a* = 7.0877 (2) Å
*b* = 8.2400 (4) Å
*c* = 21.2752 (7) Å
β = 92.659 (3)°
*V* = 1241.19 (8) Å^3^
*Z* = 4
Mo *K*α radiation
μ = 0.10 mm^−1^
*T* = 293 K
0.22 × 0.15 × 0.12 mm

#### Data collection

Bruker SMART CCD area-detector diffractometer
Absorption correction: multi-scan (*SADABS*; Sheldrick, 2004 ▶) *T*_min_ = 0.982, *T*_max_ = 0.988
11806 measured reflections
2184 independent reflections
1541 reflections with *I* > 2σ(*I*)
*R*_int_ = 0.023

#### Refinement

*R*[*F* ^2^ > 2σ(*F* ^2^)] = 0.035
*wR*(*F* ^2^) = 0.098
*S* = 1.10
2184 reflections
182 parameters
H-atom parameters constrained
Δρ_max_ = 0.10 e Å^−3^
Δρ_min_ = −0.11 e Å^−3^

### 

Data collection: *SMART* (Bruker, 2001 ▶); cell refinement: *SAINT* (Bruker, 2001 ▶); data reduction: *SAINT*; program(s) used to solve structure: *SHELXS97* (Sheldrick, 2008 ▶); program(s) used to refine structure: *SHELXL97* (Sheldrick, 2008 ▶); molecular graphics: *ORTEP-3* (Farrugia, 1997 ▶); software used to prepare material for publication: *SHELXL97*.

## Supplementary Material

Crystal structure: contains datablocks I, global. DOI: 10.1107/S1600536810038407/wn2411sup1.cif

Structure factors: contains datablocks I. DOI: 10.1107/S1600536810038407/wn2411Isup2.hkl

Additional supplementary materials:  crystallographic information; 3D view; checkCIF report

## Figures and Tables

**Table 1:** Hydrogen-bond geometry (Å, °)

*D*—H⋯*A*	*D*—H	H⋯*A*	*D*⋯*A*	*D*—H⋯*A*
O5—H5⋯N2	0.82	1.90	2.6152 (16)	145
C9—H9⋯O4	0.93	2.24	2.8937 (19)	127

**Fig. 1.**
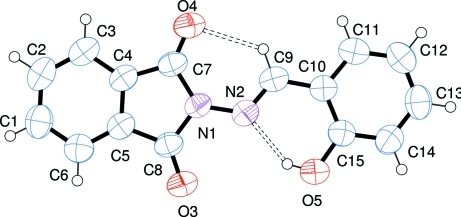
The title molecule with the displacement ellipsoids drawn at the 50% probability level. The H atoms are shown as spheres of arbitrary radius. Dashed lines indicate intramolecular hydrogen bonds.

**Fig. 2.**
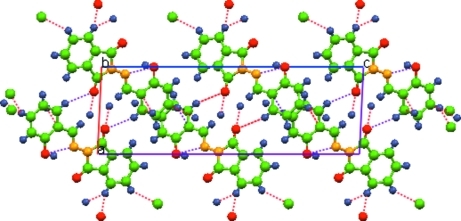
A view of the crystal structure down the b axis. Dashed lines indicate intramolecular hydrogen bonds.

## supplementary crystallographic information

### Comment

Based on the multiple binding sites of acetylcholinesterase (AChE), a series of AChE inhibitors involving phthalimide derivatives were designed and synthesized (Zhao *et al.,* 2009). Phthalimide derivatives have also been developed as LXRa-selective antagonists (Motoshima *et al.,* 2009). Azomethine group-containing compounds, typically known as Schiff bases, form a significant class of compounds in medicinal and pharmaceutical chemistry, with several biological applications that include antibacterial (Sithambaram Karthikeyan *et al.,* 2006), antifungal (Singh *et al.,* 2006) and antitumor activity (Walsh *et al.,* 1996).

The asymmetric unit of 2-{[(1E)-(2-hydroxyphenyl)methylene]amino} -1*H*-isoindole-1,3(2*H*)-dione contains one molecule (Fig. 1). The isoindoline ring system is almost planar [maximum deviation = 0.020 (2) Å] and makes a dihedral angle of 1.57 (7)° with the benzene ring. The bond distances and bond angles are in good agreement with those in a closely related crystal structure (Jing *et al.,* 2007).

In the crystal structure, molecules intramolecula O5—H5···N2 and C9—H9···O4 hydrogen bonds are observed (Table 1). The packing of the molecules shows stacking when viewed along the *b* axis (Fig. 2).

### Experimental

Salicylaldehyde (1.221 g, 10 mmol) was dissolved in 20 ml ethanol and added, with continuous stirring, to a hot ethanolic solution (30 ml) containing *N*-aminophthalimide (1.621 g, 10 mmol). The mixture was further stirred, refluxed for 5 h and allowed to stand overnight. The final yellow product was dissolved in ethanol and a single crystal of the title compound was obtained after slow evaporation of the solvent at room temperature (Yield: 89%; M.p. 465 K).

### Refinement

All H atoms were positioned at calculated positions with O—H = 0.82 Å, Csp^2^—H = 0.93 Å and refined using a riding model; *U*_iso_(H) = k*U*_eq_(attached atom), where k = 1.2 for Csp^2^ and 1.5 for O.

### Crystal data

**Table:**

C_15_H_10_N_2_O_3_	*F*(000) = 552
*M**_r_* = 266.25	*D*_x_ = 1.425 Mg m^−^^3^
Monoclinic, *P*2_1_/*c*	Melting point: 465 K
Hall symbol: -P 2ybc	Mo *K*α radiation, λ = 0.71073 Å
*a* = 7.0877 (2) Å	Cell parameters from 2184 reflections
*b* = 8.2400 (4) Å	θ = 2.7–25.0°
*c* = 21.2752 (7) Å	µ = 0.10 mm^−^^1^
β = 92.659 (3)°	*T* = 293 K
*V* = 1241.19 (8) Å^3^	Plate, yellow
*Z* = 4	0.22 × 0.15 × 0.12 mm

### Data collection

**Table:**

Bruker SMART CCD area-detector diffractometer	2184 independent reflections
Radiation source: fine-focus sealed tube	1541 reflections with *I* > 2σ(*I*)
graphite	*R*_int_ = 0.023
ω and φ scans	θ_max_ = 25.0°, θ_min_ = 2.7°
Absorption correction: multi-scan (*SADABS*; Sheldrick, 2004)	*h* = −8→8
*T*_min_ = 0.982, *T*_max_ = 0.988	*k* = −9→9
11806 measured reflections	*l* = −25→25

### Refinement

**Table:**

Refinement on *F*^2^	Secondary atom site location: difference Fourier map
Least-squares matrix: full	Hydrogen site location: inferred from neighbouring sites
*R*[*F*^2^ > 2σ(*F*^2^)] = 0.035	H-atom parameters constrained
*wR*(*F*^2^) = 0.098	*w* = 1/[σ^2^(*F*_o_^2^) + (0.0449*P*)^2^ + 0.0801*P*] where *P* = (*F*_o_^2^ + 2*F*_c_^2^)/3
*S* = 1.10	(Δ/σ)_max_ < 0.001
2184 reflections	Δρ_max_ = 0.10 e Å^−^^3^
182 parameters	Δρ_min_ = −0.11 e Å^−^^3^
0 restraints	Extinction correction: *SHELXL97* (Sheldrick, 2008), Fc^*^=kFc[1+0.001xFc^2^λ^3^/sin(2θ)]^-1/4^
Primary atom site location: structure-invariant direct methods	Extinction coefficient: 0.0088 (16)

### Special details

**Table:**

Experimental. IR ν(Nujolmull, cm^-1^): 1768 and 1725 (C=O), 1617 (HC=N), 3386 (Ph—OH). ^1^H NMR (DMSO-*d_6_*, p.p.m.): 10.67 (s, 1H, Ph—OH), 9.45 (s, 1H, HC=N), 6.92–7.91 (m, 4H, Ar—H). FAB-MS: m/*z* = 267 [*M*+1]^+^, Anal. Calc. for C_15_H_10_N_2_O_3_; C, 67.67; H, 3.79; N, 10.52; O, 18.03; Found: C, 67.05; H, 3.81; N, 10.68; O, 17.98,; Electronic spectra: 289 nm and 334 nm.
Geometry. All s.u.'s (except the s.u. in the dihedral angle between two l.s. planes) are estimated using the full covariance matrix. The cell s.u.'s are taken into account individually in the estimation of s.u.'s in distances, angles and torsion angles; correlations between s.u.'s in cell parameters are only used when they are defined by crystal symmetry. An approximate (isotropic) treatment of cell s.u.'s is used for estimating s.u.'s involving l.s. planes.
Refinement. Refinement of *F*^2^ against ALL reflections. The weighted *R*-factor *wR* and goodness of fit *S* are based on *F*^2^, conventional *R*-factors *R* are based on *F*, with *F* set to zero for negative *F*^2^. The threshold expression of *F*^2^ > 2σ(*F*^2^) is used only for calculating *R*-factors(gt) *etc*. and is not relevant to the choice of reflections for refinement. *R*-factors based on *F*^2^ are statistically about twice as large as those based on *F*, and *R*- factors based on ALL data will be even larger.

### Fractional atomic coordinates and isotropic or equivalent isotropic displacement parameters (Å^2^)

**Table:**

	*x*	*y*	*z*	*U*_iso_*/*U*_eq_	
O3	−0.27271 (15)	0.27296 (16)	0.07498 (5)	0.0836 (4)	
O4	0.28488 (17)	0.31121 (18)	−0.02433 (5)	0.0877 (4)	
O5	0.00226 (15)	0.52524 (17)	0.19986 (5)	0.0846 (4)	
H5	−0.0194	0.4752	0.1670	0.127*	
N1	0.02621 (17)	0.31346 (16)	0.03847 (5)	0.0579 (4)	
N2	0.08319 (18)	0.40089 (16)	0.09127 (5)	0.0591 (4)	
C1	−0.3130 (3)	0.0248 (2)	−0.11364 (9)	0.0780 (5)	
H1	−0.4146	−0.0282	−0.1338	0.094*	
C2	−0.1434 (3)	0.0318 (2)	−0.14198 (8)	0.0798 (5)	
H2	−0.1320	−0.0162	−0.1812	0.096*	
C3	0.0112 (3)	0.1090 (2)	−0.11336 (8)	0.0739 (5)	
H3	0.1262	0.1138	−0.1327	0.089*	
C4	−0.0109 (2)	0.1786 (2)	−0.05531 (7)	0.0580 (4)	
C5	−0.1815 (2)	0.1703 (2)	−0.02662 (7)	0.0578 (4)	
C6	−0.3351 (2)	0.0955 (2)	−0.05535 (8)	0.0716 (5)	
H6	−0.4506	0.0923	−0.0363	0.086*	
C7	0.1244 (2)	0.2722 (2)	−0.01510 (7)	0.0614 (4)	
C8	−0.1610 (2)	0.2551 (2)	0.03459 (7)	0.0607 (4)	
C9	0.2541 (2)	0.4496 (2)	0.09978 (7)	0.0610 (4)	
H9	0.3422	0.4266	0.0700	0.073*	
C10	0.3099 (2)	0.54030 (19)	0.15573 (7)	0.0557 (4)	
C11	0.4953 (2)	0.5961 (2)	0.16350 (8)	0.0700 (5)	
H11	0.5798	0.5748	0.1324	0.084*	
C12	0.5558 (3)	0.6816 (2)	0.21590 (9)	0.0783 (5)	
H12	0.6801	0.7176	0.2204	0.094*	
C13	0.4304 (3)	0.7134 (2)	0.26175 (9)	0.0781 (5)	
H13	0.4705	0.7720	0.2973	0.094*	
C14	0.2476 (3)	0.6602 (2)	0.25587 (8)	0.0741 (5)	
H14	0.1648	0.6823	0.2874	0.089*	
C15	0.1854 (2)	0.5737 (2)	0.20321 (8)	0.0610 (4)	

### Atomic displacement parameters (Å^2^)

**Table:**

	*U*^11^	*U*^22^	*U*^33^	*U*^12^	*U*^13^	*U*^23^
O3	0.0731 (8)	0.1092 (11)	0.0704 (7)	−0.0031 (7)	0.0216 (6)	−0.0101 (7)
O4	0.0713 (8)	0.1189 (12)	0.0746 (8)	−0.0043 (7)	0.0206 (6)	−0.0070 (7)
O5	0.0675 (8)	0.1089 (11)	0.0789 (8)	−0.0129 (7)	0.0191 (6)	−0.0184 (7)
N1	0.0613 (8)	0.0631 (9)	0.0498 (7)	0.0052 (6)	0.0078 (6)	0.0018 (7)
N2	0.0640 (9)	0.0584 (9)	0.0550 (8)	0.0062 (7)	0.0045 (6)	0.0027 (6)
C1	0.0967 (14)	0.0658 (13)	0.0705 (11)	0.0020 (10)	−0.0077 (10)	−0.0015 (10)
C2	0.1067 (15)	0.0726 (14)	0.0597 (10)	0.0146 (11)	0.0006 (10)	−0.0049 (9)
C3	0.0871 (12)	0.0750 (13)	0.0606 (10)	0.0132 (10)	0.0139 (9)	0.0029 (9)
C4	0.0706 (10)	0.0528 (10)	0.0510 (8)	0.0114 (8)	0.0071 (7)	0.0108 (8)
C5	0.0692 (10)	0.0525 (10)	0.0520 (8)	0.0090 (8)	0.0047 (7)	0.0111 (7)
C6	0.0745 (11)	0.0693 (12)	0.0711 (11)	0.0009 (9)	0.0043 (9)	0.0073 (10)
C7	0.0654 (10)	0.0640 (12)	0.0558 (9)	0.0106 (9)	0.0127 (8)	0.0093 (8)
C8	0.0641 (10)	0.0632 (12)	0.0556 (9)	0.0072 (8)	0.0105 (8)	0.0083 (8)
C9	0.0626 (10)	0.0622 (11)	0.0589 (9)	0.0106 (8)	0.0100 (7)	0.0092 (8)
C10	0.0587 (9)	0.0502 (10)	0.0581 (9)	0.0058 (7)	0.0031 (7)	0.0097 (7)
C11	0.0626 (11)	0.0696 (13)	0.0781 (11)	0.0025 (9)	0.0079 (8)	0.0107 (10)
C12	0.0713 (11)	0.0699 (13)	0.0928 (14)	−0.0089 (9)	−0.0062 (10)	0.0072 (11)
C13	0.0924 (14)	0.0607 (13)	0.0797 (12)	−0.0031 (10)	−0.0130 (10)	−0.0018 (10)
C14	0.0827 (12)	0.0714 (13)	0.0687 (11)	0.0012 (10)	0.0076 (9)	−0.0059 (10)
C15	0.0612 (10)	0.0568 (11)	0.0653 (10)	0.0005 (8)	0.0048 (8)	0.0043 (8)

### Geometric parameters (Å, °)

**Table:**

O3—C8	1.2041 (17)	C4—C7	1.473 (2)
O4—C7	1.2068 (17)	C5—C6	1.370 (2)
O5—C15	1.3568 (18)	C5—C8	1.479 (2)
O5—H5	0.8200	C6—H6	0.9300
N1—N2	1.3791 (17)	C9—C10	1.445 (2)
N1—C7	1.4044 (19)	C9—H9	0.9300
N1—C8	1.4105 (19)	C10—C11	1.395 (2)
N2—C9	1.2811 (18)	C10—C15	1.399 (2)
C1—C2	1.371 (2)	C11—C12	1.371 (2)
C1—C6	1.386 (2)	C11—H11	0.9300
C1—H1	0.9300	C12—C13	1.375 (2)
C2—C3	1.384 (2)	C12—H12	0.9300
C2—H2	0.9300	C13—C14	1.368 (2)
C3—C4	1.377 (2)	C13—H13	0.9300
C3—H3	0.9300	C14—C15	1.383 (2)
C4—C5	1.381 (2)	C14—H14	0.9300
			
C15—O5—H5	109.5	N1—C7—C4	105.31 (13)
N2—N1—C7	130.54 (13)	O3—C8—N1	124.36 (15)
N2—N1—C8	117.71 (11)	O3—C8—C5	130.20 (15)
C7—N1—C8	111.74 (13)	N1—C8—C5	105.43 (12)
C9—N2—N1	121.21 (13)	N2—C9—C10	119.96 (14)
C2—C1—C6	120.94 (17)	N2—C9—H9	120.0
C2—C1—H1	119.5	C10—C9—H9	120.0
C6—C1—H1	119.5	C11—C10—C15	118.16 (15)
C1—C2—C3	121.24 (17)	C11—C10—C9	119.24 (15)
C1—C2—H2	119.4	C15—C10—C9	122.60 (14)
C3—C2—H2	119.4	C12—C11—C10	121.56 (16)
C4—C3—C2	117.71 (16)	C12—C11—H11	119.2
C4—C3—H3	121.1	C10—C11—H11	119.2
C2—C3—H3	121.1	C11—C12—C13	119.14 (17)
C3—C4—C5	120.97 (16)	C11—C12—H12	120.4
C3—C4—C7	129.82 (15)	C13—C12—H12	120.4
C5—C4—C7	109.20 (13)	C14—C13—C12	120.96 (18)
C6—C5—C4	121.26 (15)	C14—C13—H13	119.5
C6—C5—C8	130.43 (14)	C12—C13—H13	119.5
C4—C5—C8	108.30 (14)	C13—C14—C15	120.29 (17)
C5—C6—C1	117.88 (16)	C13—C14—H14	119.9
C5—C6—H6	121.1	C15—C14—H14	119.9
C1—C6—H6	121.1	O5—C15—C14	117.54 (14)
O4—C7—N1	125.03 (16)	O5—C15—C10	122.57 (15)
O4—C7—C4	129.65 (15)	C14—C15—C10	119.88 (15)
			
C7—N1—N2—C9	−5.3 (2)	C7—N1—C8—O3	178.98 (14)
C8—N1—N2—C9	175.89 (14)	N2—N1—C8—C5	178.99 (12)
C6—C1—C2—C3	0.2 (3)	C7—N1—C8—C5	−0.04 (17)
C1—C2—C3—C4	0.1 (3)	C6—C5—C8—O3	3.4 (3)
C2—C3—C4—C5	0.4 (2)	C4—C5—C8—O3	−177.93 (16)
C2—C3—C4—C7	−177.72 (16)	C6—C5—C8—N1	−177.66 (16)
C3—C4—C5—C6	−1.3 (2)	C4—C5—C8—N1	1.02 (17)
C7—C4—C5—C6	177.25 (15)	N1—N2—C9—C10	−179.69 (13)
C3—C4—C5—C8	179.92 (15)	N2—C9—C10—C11	−178.34 (14)
C7—C4—C5—C8	−1.58 (17)	N2—C9—C10—C15	2.3 (2)
C4—C5—C6—C1	1.5 (2)	C15—C10—C11—C12	−0.1 (2)
C8—C5—C6—C1	−179.97 (16)	C9—C10—C11—C12	−179.50 (15)
C2—C1—C6—C5	−1.0 (3)	C10—C11—C12—C13	−0.2 (3)
N2—N1—C7—O4	−1.0 (3)	C11—C12—C13—C14	0.4 (3)
C8—N1—C7—O4	177.88 (15)	C12—C13—C14—C15	−0.3 (3)
N2—N1—C7—C4	−179.74 (14)	C13—C14—C15—O5	−179.50 (15)
C8—N1—C7—C4	−0.87 (17)	C13—C14—C15—C10	0.0 (3)
C3—C4—C7—O4	1.2 (3)	C11—C10—C15—O5	179.69 (14)
C5—C4—C7—O4	−177.15 (17)	C9—C10—C15—O5	−0.9 (2)
C3—C4—C7—N1	179.85 (16)	C11—C10—C15—C14	0.2 (2)
C5—C4—C7—N1	1.52 (17)	C9—C10—C15—C14	179.56 (15)
N2—N1—C8—O3	−2.0 (2)		

### Hydrogen-bond geometry (Å, °)

**Table:**

*D*—H···*A*	*D*—H	H···*A*	*D*···*A*	*D*—H···*A*
O5—H5···N2	0.82	1.90	2.6152 (16)	145
C9—H9···O4	0.93	2.24	2.8937 (19)	127
